# Increased expression of SET domain-containing proteins and decreased expression of Rad51 in different classes of renal cell carcinoma

**DOI:** 10.1042/BSR20160122

**Published:** 2016-06-30

**Authors:** Si Liu, Yiyang Li, Hongmei Xu, Kaichen Wang, Nan Li, Jia Li, Tao Sun, Ying Xu

**Affiliations:** * Department of Urology, The First Hospital of Jilin University, Jilin 130021, China; † Department of Gynaecology, The First Hospital of Jilin University, Jilin 130021, China; ‡ Department of Obstetrics, The First Hospital of Jilin University, Jilin 130021, China; § Department of Urology, The Third Hospital of Jilin University, Jilin 130031, China; ║ Intensive Care Unit, The First Hospital of Jilin University, Jilin 130021, China; ¶ Department of Nephrology, The First Hospital of Jilin University, Jilin 130021, China

**Keywords:** epigenetic, genome instability, histone, metastasis, methyltransferase, renal cell cancer

## Abstract

In the present study, we aimed to examine whether SET domain-containing methyltransferases are up-regulated in different classes of renal cell carcinoma. We immunoblotted against SET domain and quantified the expression of these modular domains. Furthermore, we examined the expression of Rad51, the key protein that confers genomic stability. There was enhanced expression of SET domain-containing histone methyltransferases in whole lysates of all classes of renal carcinoma. In metastatic high grade clear cell carcinoma, this expression was more pronounced. Though we could not demonstrate direct correlation, we showed that epigenetic modification by methylation is associated with decreased genomic translation of Rad51.

## Abstract (precis)

Because of scant availability of tissue samples, we did not perform elaborate examination of chromatin immunoprecipitation and specific binding of SET domain-containing proteins to the promoters of Rad51. These remain avenues for future investigations.

## INTRODUCTION

Post-translational modifications of N-terminal histone by methylation can bring about significant epigenetic changes [1–5]. These non-inheritable changes have significant impacts on the course of cancerous lesions by affecting gene transcription in a positive and/or negative way [5]. One of the constant findings in renal cell carcinoma involves detection of different grades of methylation, including mono-, di or trimethylation [6–8].

Histone methylation occurs at arginine and lysine residues of the tail zones of histone H3 and H4 [9]. Coactivator-associated arginine methyltransferase 1 (CARM1) and protein arginine *N*-methyltransferase 1 (PRMT1) are key histone arginine methylases that possess an *S*-adenosyl-L-methionine (AdoMet) motif [10]. These histone arginine methylases, however, lack ability to methylate other amino acid residues such as lysine [11].

It has been demonstrated that renal cell carcinoma shows significant lysine methylation [6–8]. Lysine methylation is carried out by a novel class of enzymes that contains the modular protein SET domain. The first SET domain-containing histone lysine methylase that was reported is the mammalian Suv39h1, which adds methyl group to histone H3 at Lys-9 [12]. Several SET domain-containing proteins, namely Set1, Set2, Set7/Set9, G9a and ESET, have now been shown to methylate numerous lysine residues including Lys-4, Lys-9, Lys-27 or Lys-36 of histone H3 tail [13–15].

Renal cell carcinoma has been reported to be methylated at lysine residues of histones [6–8]. In the present study, we aimed to examine whether SET domain-containing methyltransferases are up-regulated in different classes of renal cell carcinoma. In order to detect these enzymes, we immunoblotted against SET domain and quantified the expression of these modular domains. Furthermore, we examined the expression for Rad51, the key protein that confers genomic stability [16]. We consistently observed inverse relationships between the expression of SET domain-containing proteins and the expression of Rad51 in different classes of renal carcinoma.

## MATERIALS AND METHODS

Studies were performed after obtaining explicit consent from patient families, institutional IRB permission for conducting studies with human tissues, and completely in strict adherence to Helsinki guidelines.

### Microscopic identification of renal cancer tissues

Fresh tissue sections were obtained from surgical samples (*n*=15 in each group, both males and females, age range 24–80 years), including low grade renal clear cell carcinoma, high grade metastatic renal cell carcinoma (confirmed from history), chromophobe carcinoma, papillary carcinoma and normal renal tissues. Sections were stained with Giemsa and the diagnosis was confirmed based on nuclear morphology by Fuhrman classification. Five randomly selected patients or subjects samples were pooled randomly, thus generating three independent sets of samples (triplicates). All triplicate samples were examined for markers of histone methyltransferase activities. This approach was adapted to ensure variability in the biological samples and to examine the trend, given the small sample size.

### Antibodies and chemicals

Numerous pilot experiments were conducted to assay the antibody as well to optimize its concentration for immunodetection during western blotting. For all experiments, control experiments were performed by eliminating the use of primary and secondary antibodies respectively. Antibodies were obtained from Santa Cruz Biotechnology. All chemicals were obtained from Sigma–Aldrich.

### Isolation of nuclear fraction

Tissues from cores of cancer masses were washed in PBS and suspended in 1 ml of PBS in 1.5 ml microfuge tubes and initially spun for 30 s at 1000 ***g***. Thereafter, the cell suspensions were incubated in NP-40 (Calbiochem) and triturated repeatedly with a micropipette, and centrifuged for 15 min at 3000 ***g***. The supernatant, the cytosolic fraction, was decanted, and the pellet fraction was washed once with NP-40-PBS, and recentrifuged for five more minutes. The pelted fragment, representative of the nuclear fraction, was carefully collected and stored in the refrigerator until further examination.

### Real-time PCR

Total RNA was extracted after homogenization of cells and tissues using RNeasy mini kit (Qiagen Sciences). Total RNA (1 μg) was reverse transcribed with the High Capacity cDNA Reverse Transcription Kit (Applied Biosystems). The cDNA reaction was diluted to 1:10 for use as the template for real-time RT-PCR. For quantitative real-time PCRs (qPCRs), TaqMan Gene Expression Assays primers and probes specific to Rad51 or mixed lineage leukaemia 5 (MLL5) were used for expression analyses and GAPDH primers and probes (Applied Biosystems) were used as internal controls. Analyses were performed using the MX400 Multiplex Quantitative PCR system (Stratagene). The cycling conditions were as follows: one cycle of 2 min at 50°C, one cycle of 10 min at 95°C, 40 cycles of denaturation (15 s at 95°C) and annealing/extension (1 min at 60°C). All quantitative PCR reactions were carried out in triplicate and repeated at least twice. The ΔCt for mRNA expression was calculated relative to the Ct (threshold cycle) of GAPDH mRNA. Relative mRNA expression was calculated using the formula 2 (-ΔΔCt). Primers and probes for all analyses are available upon request.

### Western blotting

Protein lysates were prepared from dissected cellular mass using lysis buffer [1% (v/v) Triton X-100 and 1% (v/v) NP-40 (Calbiochem), dissolved in deionized PBS], and supplemented with proteases inhibitors cocktail (Roche Diagnostics) for half an hour on ice bucket. Cell lysates were mixed with a vortex and centrifuged at 20,000 rpm at 4°C for 15 min. Supernatants were collected and protein concentration was confirmed using the Bradford assay (BioRad Protein Assay kit, USA) prior to loading gels. Proteins were added to sample buffer [Laemmli with 5% (v/v) 2-β-mercaptoethanol and 5% (v/v) bromophenol blue] and boiled for 5 min at 100°C. Samples were subjected to sodium dodecyl sulfate-polyacrylamide gel electrophoresis (SDS-PAGE) at 100 V and proteins were transferred onto polyvinylidene fluoride (PVDF) membranes overnight in the cold and visualized by enhanced chemiluminescence (ECL). For immunostaining, membranes were blocked with 5% (w/v) non-fat dry milk in PBS containing 0.5% (v/v) Tween 20 and incubated for an hour with specific primary antibodies. After washing with PBS-Tween 20, membranes were incubated with horseradish peroxidase (HRP)-conjugated specific secondary antibodies (Santa Cruz Biotechnology), usually diluted at a 10-fold level (in comparison with the primary) for an hour. Proteins were then detected using ECL reagent (GE Healthcare Life Sciences) as a substrate prior to developing with X-ray in a dark room. For assay of loading controls, membranes were stripped using a mild protocol of washing and reprobed with the housekeeping protein GAPDH.

### Quantification of image intensities

The gels were scanned with Metamorph image analysis software without any alteration to the gamma settings. Image intensities were surrogates as reflective of protein expression levels and means were compared.

### Statistical analyses

Data were analysed using ANOVA and considered statistically significant when the P values were less than 0.05. Statistical analyses were performed using Office Excel 2010.

## RESULTS

### Increased expression of SET3 domain-containing methyltransferase in whole lysates of all classes of renal carcinoma

**Figure 1:**
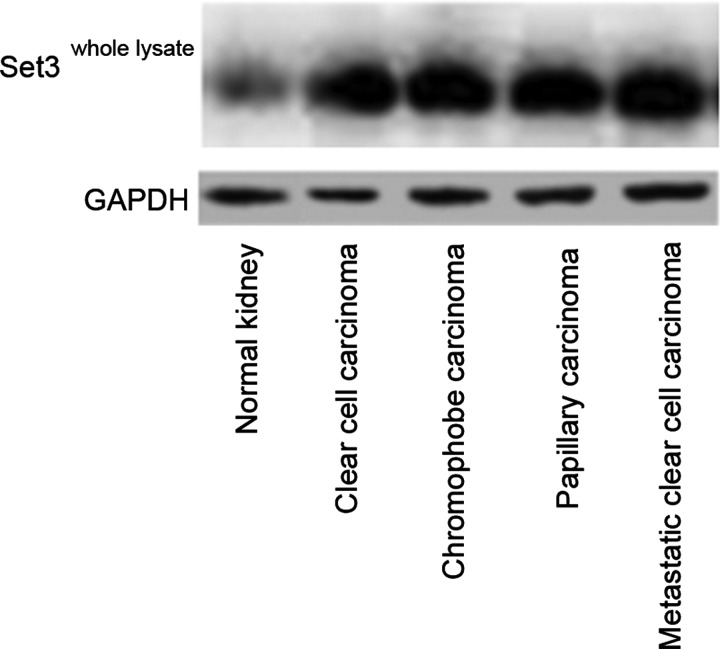
Increased expression of SET domain-containing proteins in whole lysates of different classes of renal cell carcinoma in comparison with control renal tissues Representative western blots are shown. Quantitative estimates showed highly significant difference between the means (*P*<0.0001, ANOVA, Tukey's HSD post-hoc test).

In comparison with control renal tissues, all classes of renal carcinoma, namely clear cell carcinoma, chromophobe carcinoma, papillary carcinoma and metastatic clear cell carcinoma, showed enhanced expression of monomethyl-transferase enzymes containing SET3 domains. These proteins are the major classes of enzymes that facilitate transfer of one to several methyl residues on lysines in different classes of histones including H3. Representative western blots of the whole lysates are shown in Figure 1. Quantitative estimates of protein expression showed high levels of difference when the means were compared between the different groups (1.46±0.12 compared with 3.21±0.48 compared with 2.49±0.38 compared with 2.91±0.23 compared with 3.78±0.34, control renal tissues compared with clear cell carcinoma compared with chromophobe carcinoma compared with papillary carcinoma compared with metastatic clear cell carcinoma respectively, *P*<0.001, ANOVA).

### Increased expression of SET3 domain-containing methyltransferase in nuclear extracts of all classes of renal carcinoma

**Figure 2:**
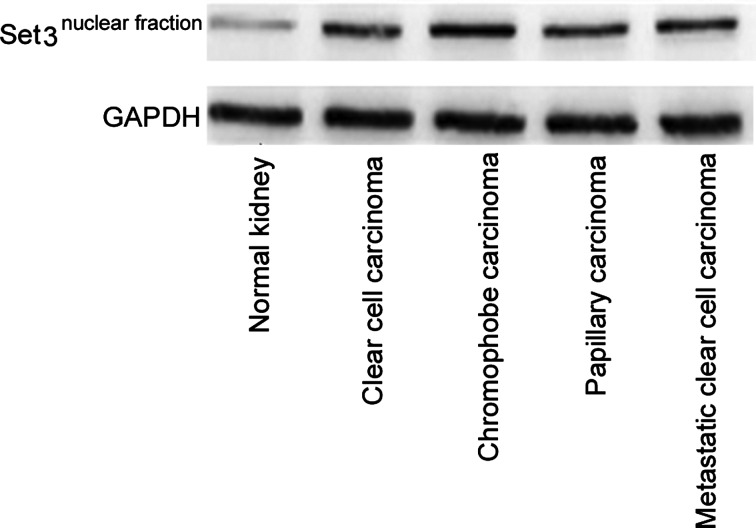
Increased expression of SET domain-containing proteins in nuclear fractions of different classes of renal cell carcinoma in comparison with control renal tissues Representative western blots are shown. Nuclear fractions were eluted by differential centrifugation. Quantitative estimates showed highly significant difference between the means (*P*<0.0001, ANOVA, Tukey's HSD post-hoc test).

In comparison with control renal tissues, all classes of renal carcinoma, namely clear cell carcinoma, chromophobe carcinoma, papillary carcinoma and metastatic clear cell carcinoma, showed enhanced expression of monomethyl-transferase enzymes containing SET3 domains within the nuclei, indicating significant presence of histone monomethyl transferases within the nuclei of carcinomatous tissues. Representative western blots of the nuclear extracts are shown in Figure 2. Quantitative estimates of protein expression were as follows: (1.21±0.07 compared with 2.71±0.18 compared with 2.56±0.24 compared with 2.55±0.12 compared with 2.81±0.29, control renal tissues compared with clear cell carcinoma compared with chromophobe carcinoma compared with papillary carcinoma compared with metastatic clear cell carcinoma respectively, *P*<0.001, ANOVA).

### Decreased expression of Rad51 in all classes of renal carcinoma

**Figure 3:**
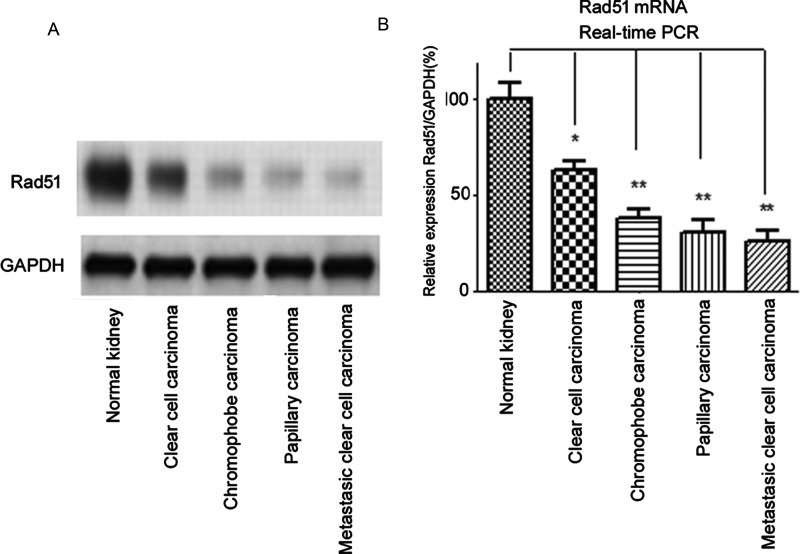
Decreased expression of Rad51 in whole lysates of different classes of renal cell carcinoma in comparison with control renal tissues (**A**) Representative western blots are shown. Quantitative estimates showed highly significant difference between the means (*P*<0.0001, ANOVA, Tukey's HSD post-hoc test). (**B**) The mRNA expressions of Rad51 were analysed by real-time PCR. Columns, mean of three independent experiments; bars, S.E. *, *P*<0.05; **, *P*<0.01.

In comparison with control renal tissues, all classes of renal carcinoma, namely clear cell carcinoma, chromophobe carcinoma, papillary carcinoma and metastatic clear cell carcinoma, showed significantly decreased expression of Rad51 which plays a major role in maintaining genomic stability. Representative western blots of the whole lysates are shown in Figure 3(A). Quantitative estimates of protein expression showed high levels of difference when the means were compared between the different groups (1.12±0.09 compared with 0.51±0.03 compared with 0.43±0.03 compared with 0.4±0.02 compared with 0.38±0.05, control renal tissues compared with clear cell carcinoma compared with chromophobe carcinoma compared with papillary carcinoma compared with metastatic clear cell carcinoma respectively, *P*<0.001, ANOVA). To assess the mRNA levels of Rad51 in normal renal tissue and tumours, we performed RT-PCR experiments to measure the mRNA expression of Rad51 using the same tissue samples in western blot experiments (Figure 3A). Consistently, we found the mRNA expression of Rad51 was significantly down-regulated in renal tumours (clear cell carcinoma, chromophobe carcinoma, papillary carcinoma and metastatic clear cell carcinoma) compared with human normal renal tissues (Figure 3B).

### Unaltered levels of expression of mixed lineage leukaemia 5 in all classes of renal carcinoma

**Figure 4:**
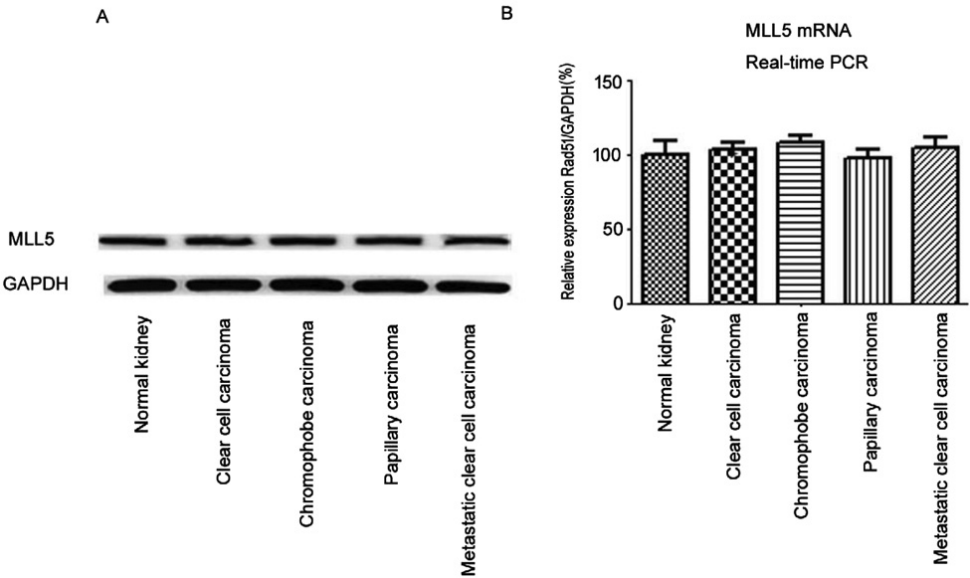
Unaltered expression of another methyltransferase, MLL5, in whole lysates of different classes of renal cell carcinoma and control renal tissues (**A**) Representative western blots are shown. Quantitative estimates showed no significant difference between the means (*P*>0.05, ANOVA, Tukey's HSD post-hoc test). (**B**) The mRNA expressions of MLL5 were analysed by real-time PCR. Columns, mean of three independent experiments; bars, S.E.

All classes of renal carcinoma, namely clear cell carcinoma, chromophobe carcinoma, papillary carcinoma and metastatic clear cell carcinoma, showed similar expression levels of MLL5 to control renal tissues. Representative western blots of the whole lysates are shown in Figure 4(A). Quantitative estimates of protein expression were as follows (1.42±0.04 compared with 1.51±0.12 compared with 1.48±0.22 compared with 1.4±0.23 compared with 1.38±0.35, control renal tissues compared with clear cell carcinoma (CCC) compared with chromophobe carcinoma compared with papillary carcinoma compared with metastatic CCC respectively, *P*>0.05, ANOVA). Moreover, the mRNA expression of MLL5 was not altered in renal tumours compared with normal renal tissue (Figure 4B).

## DISCUSSION

The results of the present study show that renal cancer cells have the potential to modify histone core domains to regulate nuclear processes. Specifically, we demonstrate that up-regulation of SET domain is associated with down-regulation of expression of Rad51, a key regulator of genomic stability [16]. Rad51 plays an important role in DNA repair [17–19]. We first demonstrated enhanced expression of SET domain-containing histone methyltransferases in whole lysates of all classes of renal carcinoma, namely clear cell carcinoma, chromophobe carcinoma and papillary carcinoma. In metastatic high grade clear cell carcinoma, this expression was more pronounced. This was also the condition when we specifically examined the translocation of these proteins in the separated nuclear fraction. We did not examine sarcomatoid variants of renal cell carcinoma due to lack of availability of adequate samples in our case series.

To examine whether there is a global up-regulation of histone methyltransferases, we examined the expression of MLL5, a methyltransferase that is increased in expression in many leukaemic conditions [20]. MLL5 expression remained unaltered, and is comparable to the control renal tissues in all classes of examined renal carcinoma tissues.

The SET domain is a conserved 130–150 amino acids protein span which was originally identified as a common element in chromatin regulators with different activities: the suppressor of position-effect variegation, Su(var)3-9, the PcG protein Enhancer of Zeste [E(z)], and trithorax (TRX) [21]. It has been demonstrated that SUV39H1, the mammalian homologue of Su(var)3–9, methylates lysine 9 of histone H3 [12,21]. This modification creates a binding site for HP1 and thus can contribute to the progress of a heterochromatin domain [22,23]. Whereas the histone–methylase activity of SUV39H1 was critically dependent on the SET domain, additional protein domains were also required.

Preliminary evidence has provided support for this hypothesis that the histone tails undergo distinct posttranslational modifications that may constitute a ‘histone code’ [24,25] and have influence on the binding of specific chromatin-associated proteins. Though we could not demonstrate direct correlation, we showed that epigenetic modification by methylation is associated with decreased genomic translation of Rad51, the key regulator of genomic stability [16]. Because of scant availability of tissue samples, we did not perform elaborate examination of chromatin immunoprecipitation and specific binding of SET domain-containing proteins to the promoters of Rad51. These remain avenues for future investigations.

## Abbreviations

CCC: clear cell carcinoma
ECL: enhanced chemiluminescence
MLL5: mixed lineage leukaemia 5
SDS-PAGE: sodium dodecyl sulfate-polyacrylamide gel electrophoresis
